# The Impact of Ontario’s Virtual Care Payment Model on Cancer Care: A Natural Policy Experiment

**DOI:** 10.2196/89151

**Published:** 2026-04-20

**Authors:** Adam Suleman, Zhihui Amy Liu, Anna Sinaiko, Anca Prica, Danielle Rodin

**Affiliations:** 1 Oakville-Trafalgar Memorial Hospital Oakville, ON Canada; 2 Princess Margaret Cancer Centre Toronto, ON Canada; 3 Department of Health Policy and Management Harvard University Boston, MA United States

**Keywords:** virtual care, cancer, time series, policy, payment model

## Introduction

Virtual care for oncology patients via phone or video visits significantly increased during the COVID-19 pandemic to support ongoing care delivery [1-3]. In Ontario, this was facilitated by changes in the publicly funded Ontario Health Insurance Program, through which most physicians are remunerated. From March 14, 2020, to November 30, 2022, all virtual visits were payable at the same rate as in-person care [4]. On December 1, 2022, payment for new patient consultations by phone was reduced to CAD $15 and phone follow-up visits payable at 85% of the equivalent in-person rate. We evaluated the association between the December 2022 change in payment and the use of phone visits for care delivery.

## Methods

We obtained data on all ambulatory oncology clinic visits from Princess Margaret Cancer Center (Multimedia Appendix 1), Canada’s largest comprehensive cancer center, delivered between June 1 and November 30, 2022 (period 1: prior to policy change or “interruption”), and between December 1, 2022, and May 31, 2023 (period 2: after policy change).

An interrupted time series (ITS) analysis was conducted, modeling the total number of weekly visits using a linear regression model. An outlier holiday week of December 24-30, 2022, was removed from the analysis. Model parameters included the intercept, baseline trend (preinterruption slope), step change after policy change, and trend change after policy implementation. Autocorrelation and partial autocorrelation plots and the Durbin-Watson statistic were used to examine temporal autocorrelation in the model residuals. To evaluate whether modality-specific changes might reflect broader utilization trends, individual ITS analyses for total, in-person, phone, and video visits were performed. Subgroup analyses were performed for individual clinic types, including for hematologic malignancies, radiation therapy, solid tumor, and supportive care clinics. A 2-sided *P* value <.05 was considered statistically significant. This study was approved by the University Health Network research ethics board (REB 23-5510).

## Results

There were 144,139 visits during the study (75,849 during period 1; 68,290 during period 2), of which 13% (9860/75,849) and 7% (4780/68,290) were by phone in periods 1 and 2, respectively. Video visits comprised 4% (3034/75,849) of the visits in period 1 and 12% (8195/68,290) in period 2. Visits were to outpatient clinics for solid tumors (108,620/144,139, 75%), hematologic malignancies (22,301/144,139, 15%), and supportive care (4874/144,139, 3%), and on-treatment visits for radiation therapy (8344/144,139, 6%).

**Figure 1:**
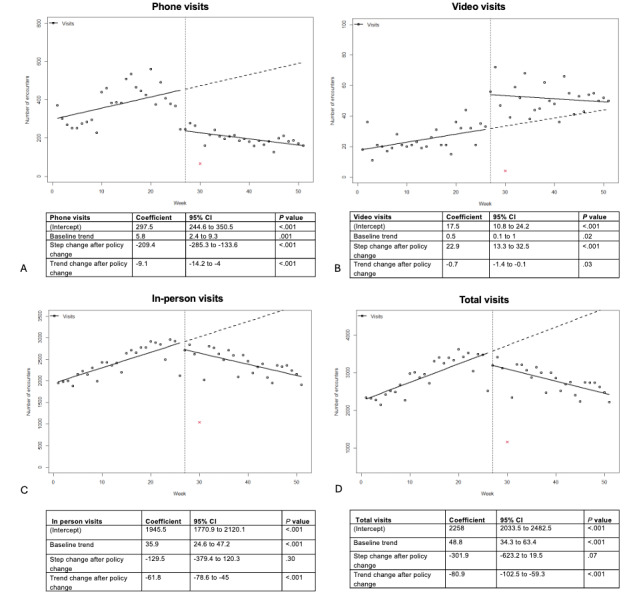
Phone visits per week (panel A), video visits per week (panel B), in-person visits per week (panel C), and total visits per week (panel D) for patients with an oncology visit from June 2022 to May 2023. x: Christmas holiday week excluded from the analysis.

Before the policy change, phone visits were increasing by 5.8 visits per week. At the time of the policy change, there was an immediate reduction of 209.4 phone visits per week (95% CI –285.3 to –133.6; *P*<.001). After the policy change, the weekly trend also reversed, with a slope change of –9.1 visits per week (95% CI –14.2 to –4; *P*<.001), corresponding to an overall post-policy decline of 3.3 phone visits per week (Figure 1A).

**Figure 2:**
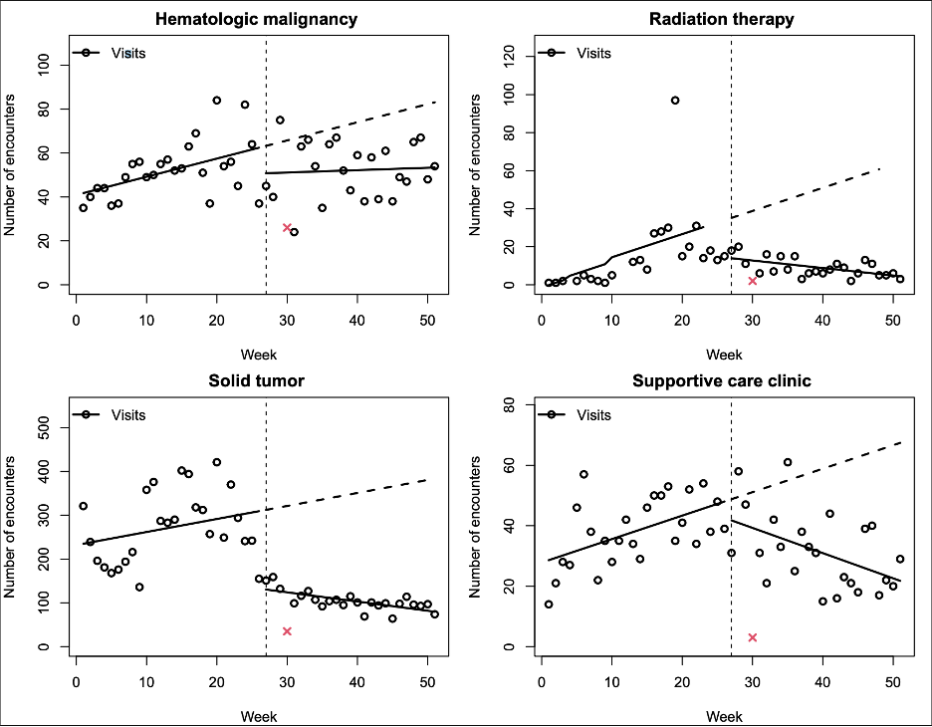
Phone visits per week by clinic type for patients with an oncology visit from June 2022 to May 2023. x: Christmas holiday week excluded from the analysis.

This decreasing trend was observed for all clinic types (Figure 2). In contrast, the number of weekly video visits immediately increased by 22.9 (95% CI 13.3-32.5; *P*<.001) at the time of the policy change compared to a baseline increasing trend of 0.5 weekly visits. After the policy change, the weekly trend reversed, with a slope change of 0.7 (95% CI –1.4 to –0.1; *P*=.03) (Figure 1B).

Unlike for virtual care, there was no significant immediate change in the number of weekly in-person (Figure 1C) or total visits (Figure 1D). After the policy change, there was a decreasing trend, consistent with the reduction observed in virtual care, with a slope change of –61.8 (95% CI –78.6 to –45; *P*<.001), corresponding to 25.9 fewer in-person visits per week in period 2 compared to those in period 1 and an overall reduction of 32.1 total visits per week between the two periods.

## Discussion

An immediate shift in visit modality was observed following the payment policy change, with a sharp drop in phone visits and a sharp increase in video visits, with no change in visits in-person. In the 6 months following, there was a general trend toward declining virtual care, reversing the initial increase in video visits following the fee code revision, along with a decline in in-person visits, suggesting an inflection point in the modality used for health care delivery at the time of the policy change. The overall reduction in visits over time suggests the contribution of broader temporal changes in care delivery.

There is ongoing tension in defining the optimal use of virtual care. Virtual care offers enhanced access to timely support and symptom management for patients with cancer, reducing the need for frequent travel during physically and emotionally challenging treatment periods [5]. In fee-for-service environments, however, financial incentives have been shown to impact physician behavior [6,7], and there is concern that physicians may feel incentivized to favor brief phone encounters over in-person visits, increasing the frequency of low‑value visits [8]. In the absence of other policies or operational changes that may have coincided with the fee change, findings from this natural policy experiment suggest that remuneration may have played a role in the immediate shift in visit modality. However, unmeasured concurrent changes in clinician behavior or patient preferences could still contribute to residual temporal confounding.

Limitations of this study include the lack of clinical or sociodemographic information, which may impact patient or physician preference for virtual care or clinic- or hospital-level factors that may impact physicians’ ability to schedule virtual visits. This study was performed in a specialized academic center where virtual care was widely offered throughout the pandemic [1,3], and the findings may not be applicable to other settings that had not routinely adopted virtual care during this period. Further, we cannot attribute causality from the policy change to the observed findings, and the gradual overall decline in visits suggest other unmeasured concurrent changes in care delivery or preferences.

The option of virtual care has been valued by many patients with cancer [9,10]; thus, policymakers should evaluate whether the current fee structure is contributing to reduced virtual care access and reduced agency in choosing virtual care when appropriate. Further studies are needed to understand how policy changes, along with patient and provider preferences, impact the modality of care delivery for patients with cancer.

## Appendix

Multimedia Appendix 1 Number of ambulatory visits to Princess Margaret Cancer Centre by appointment type (in person or virtual) and clinic type during the study period.

## Abbreviations

ITS: interrupted time series
