# Supplementary material for: The Impact of Ontario’s Virtual Care Payment Model on Cancer Care: A Natural Policy Experiment
Source: J Med Internet Res. 2026 Apr 20;28:e89151. doi: 10.2196/89151 (PMC13139831; doi:10.2196/89151)
Supplement: Multimedia Appendix 1 [file jmir_v28i1e89151_app1.docx]

**Appendix 1**

|  | **Full Sample (n=144,139)** | **Period 1 (n=75,849)** | **Period 2  (n=68,290)** |
| --- | --- | --- | --- |
| **Appointment type** |  |  |  |
| In person (n,%) | 121,848 (85) | 63,171 (83) | 58,677 (86) |
| Virtual (n,%) | 22,291 (15) | 12,678 (17) | 9,613 (14) |
| **Clinic** |  |  |  |
| Hematologic malignancy (n,%) | 22,301 (15) | 11,853 (16) | 10,448 (15) |
| Radiation therapy (n,%) | 8,344 (6) | 5,011 (7) | 3,333 (5) |
| Solid tumor (n,%) | 10,8620 (75) | 56,565 (75) | 52,055 (76) |
| Supportive care clinic (n,%) | 4,874 (3) | 2,420 (3) | 2,454 (4) |

Number of ambulatory visits to Princess Margaret Cancer Centre by appointment type (in person or virtual) and clinic type during the study period.
